# Antimicrobial Susceptibility Trends in *E. coli* Causing Pediatric Urinary Tract Infections in the United States

**DOI:** 10.3390/pathogens13121068

**Published:** 2024-12-06

**Authors:** Simren Mahajan, Neena Kanwar, Gina M. Morgan, Rodrigo E. Mendes, Brian R. Lee, Dithi Banerjee, Rangaraj Selvarangan

**Affiliations:** 1 School of Medicine, University of Missouri, Kansas City, MO 64108, USA; smahajan@cmh.edu (S.M.); dbanerjee@cmh.edu (D.B.); rselvarangan@cmh.edu (R.S.); 2 Children’s Mercy, Kansas City, MO 64108, USA; blee@cmh.edu; 3 JMI Laboratories, North Liberty, IA 52317, USA; gina.bartleson@element.com (G.M.M.); rodrigo.mendes@element.com (R.E.M.)

**Keywords:** antimicrobial susceptibility, pediatric, MIC, *E. coli*, UTI, susceptibility trends, surveillance

## Abstract

Urinary tract infections (UTIs) are among the most common pediatric infections. This study evaluated the antimicrobial susceptibility patterns of 3511 uropathogenic *E. coli* (UPEC) isolated from pediatric patients in the United States from 2014 to 2023. The database from the SENTRY antimicrobial surveillance program from 89 medical centers was utilized as a data source. The antimicrobial susceptibility was tested using the microbroth dilution technique against 24 antimicrobial agents. MICs were determined using the CLSI/EUCAST/FDA breakpoint criteria. All the antimicrobials reported susceptibility rates above 80% except for tetracycline (76.2%), trimethoprim–sulfamethoxazole (69.7%), and ampicillin–sulbactam (55.7%). During the study period, the susceptibility rates remained stable for most antimicrobial agents. However, significant differences were observed among age, gender, and U.S. census regions, with the Middle Atlantic showing the lowest and the Mountain region the highest susceptibility rates, for most antimicrobials. The incidence of ESBL UPEC increased from 7.1% to 10.8% between 2014 and 2023, while the prevalence of the MDR phenotype remained relatively stable. The prevalence of both ESBL and MDR phenotypes was highest among infants and young children (0–24 months), with the highest resistance rates from the Pacific region. Knowledge of the landscape of antibiotic resistance in pediatric UPEC will help healthcare providers to better tailor empiric treatment regimens for most UTI infections.

## 1. Introduction

Urinary tract infections (UTIs) are among the most common bacterial infections in children, with a significantly higher risk in girls, except during the first year of life in which UTIs are more prevalent in boys [1,2]. *Escherichia coli* is the predominant uropathogen responsible for 70–90% of all uncomplicated UTIs [3,4] and more than 50% of complicated UTIs [5].

Common antibiotics used for UTI treatment include nitrofurantoin, first-generation cephalosporin (e.g., cephalexin), third-generation cephalosporin, amoxicillin–clavulanate, and trimethoprim–sulfamethoxazole [4,6,7]. Increased rates of antibiotic-resistant uropathogenic *E. coli* (UPEC) poses significant challenges to the effective management of UTIs in children [4,5,8]. The resistance rates for trimethoprim–sulfamethoxazole exceed 20%, while those for amoxicillin are much higher, making it a less favorable choice for empiric treatment [8]. Oral cephalosporins also exhibit varying resistance levels, depending on the specific cephalosporin [9]. In contrast, nitrofurantoin continues to demonstrate relatively low resistance rates, making it a reliable treatment option for UTIs [10].

This study analyzed the antimicrobial susceptibility patterns of commonly used antibiotics against UPEC in children diagnosed with UTIs. Analyses by age, gender, and geographical regions were performed to highlight the implications of different demographics on antibiotic resistance patterns over the 10-year study duration. Additional analyses focused on *E. coli,* demonstrating extended-spectrum beta-lactamase (ESBL) and carbapenem-non-susceptible phenotypes, as well as multi-drug-resistant (MDR; isolates non-susceptible to ≥1 agent in ≥3 antimicrobial categories) and extensively drug-resistant (XDR; isolates non-susceptible to all almost all antimicrobial agents tested) isolates. The prevalence of all MDR/XDR *E. coli* including the ESBL-producing phenotypes has been on the rise globally, posing significant challenges in treating UTIs [9,10,11,12,13]. By understanding the landscape of antibiotic resistance in pediatric *E. coli* UTIs, healthcare providers can better tailor treatment regimens, improve patient outcomes, and contribute to broader efforts in combating antimicrobial resistance.

## 2. Materials and Methods

### 2.1. Study Design and Population

This study presents a 10-year retrospective multicenter data analysis of 3511 *E. coli* UTI isolates collected from pediatric patients from the United States (U.S.) aged 0 years to 18 years old (average age: 7.28 years). These isolates were collected from 2014 to 2023 as part of the SENTRY Antimicrobial Surveillance Program (SENTRY; https://www.jmilabs.com/sentry-surveillance-program/, accessed on 1 September 2024). SENTRY is a global surveillance system designed to collect and analyze the data on the distribution of bacterial species causing various types of clinical infections and to monitor their susceptibility to a wide range of antibiotics. This database was also utilized as a source of antimicrobial susceptibility results for this study.

Eighty-nine participating U.S. medical centers collected and shipped a target number of consecutive samples of UTI pathogens over the study duration to Element Iowa City (JMI Laboratories, North Liberty, IA, USA), which served as a central reference laboratory. A demographic form accompanied each isolate, capturing patient-specific information such as hospital admission date, age, gender, specimen type, diagnosis, date of positive culture, and infection origin (nosocomial vs. community acquired). The patient demographics are described in Table 1.

### 2.2. Bacterial Isolation and Identification

*E*. *coli* isolates were obtained from the urine samples of U.S. pediatric patients aged 0 to 18 years old with a UTI. Only isolates from urine cultures that had significant colony counts (>100,000 CFU/mL), signifying the causative agent for the UTI, qualified for inclusion in this study. All the isolates were identified and confirmed as per the individual institution’s standard-of-care procedures. Further, if necessary, confirmation of bacterial identification was performed at the reference laboratory by matrix-assisted laser desorption/ionization time-of-flight (MALDI-TOF) mass spectrometry, molecular sequencing, or biochemical tests upon arrival. Cultures with bacterial pathogens other than *E. coli* were excluded from this analysis.

### 2.3. Susceptibility Testing

Antibiotic susceptibility testing was conducted at the reference laboratory by determining the minimum inhibitory concentration (MIC) using the broth microdilution method as per the Clinical and Laboratory Standards Institute (CLSI) guidelines [14]. A panel of 24 antimicrobials covering commonly used antibiotics was tested over the 10-year study duration (Table 2). The MIC endpoints (susceptibilities) were interpreted by utilizing the CLSI 2024 breakpoint criteria for all the antimicrobials [14] except for colistin and tigecycline, for which, the EUCAST (http://www.eucast.org/clinical_breakpoints/, accessed 1 September 2024) and U.S. Food and Drug Administration (FDA; Tigecycline—Injection products|FDA) interpretive breakpoints, respectively, were used.

### 2.4. Data Analysis

The rates of susceptible isolates were determined by dividing the total number of isolates that were observed to be susceptible to each agent by the CLSI/EUCAST/FDA guidelines by the total number of isolates tested. The median MIC value was considered the MIC_50_. Similarly, MIC_90_ refers to the minimum antibiotic concentration required to inhibit 90% of the isolates tested for each agent. The data were analyzed by four different age categories, gender, nine different U.S. census regions, and resistance profile. The age distribution was classified into four different categories: infants and young (0–24 months), preschool (3–5 years), school-aged (6–12 years), and adolescent (13–18 years) children. The susceptibility rates were compared using the chi-square test.

### 2.5. Ethical Considerations

The study protocol was reviewed and approved by the institutional review boards (IRBs) of the participating hospitals and laboratories. Patient confidentiality was maintained by de-identifying all the data prior to sending isolates to the reference laboratory.

## 3. Results

The overall percent susceptibilities of UPEC to common antimicrobial agents are presented in Table 2. Several agents (ceftazidime–avibactam, ceftolozane–tazobactam, colistin, imipenem, meropenem, nitrofurantoin, piperacillin–tazobactam, and tigecycline) exhibited a high rate of activity (>95% susceptible), with ceftazidime–avibactam and meropenem demonstrating 100% susceptibility rates. Ampicillin–sulbactam, tetracycline, and trimethoprim–sulfamethoxazole were found to be the least active agents with susceptibility percentages <80.0% (Table 2).

The antimicrobial susceptibility trends of pediatric UPEC from 2014 through 2023 are presented in Table 3. Antimicrobial susceptibility for ceftazidime–avibactam, imipenem, meropenem, and tigecycline ranged yearly between 99.5% and 100% over the 10-year study period. Susceptibility percentages remained relatively stable over the years for most antibiotics, with the exception of gentamicin and tobramycin, which saw decreased activity of 6% (*p*-value = 0.26) and 4.5% (*p*-value = 0.43), respectively, in 2023 compared to 2021.

The susceptibility percentages of UPEC isolated from each age category are presented in Table 4. Significant differences between the different age categories were observed for ampicillin–sulbactam (infants and young: 52.2%; preschool: 53.6%; school-aged: 56.0%; adolescents: 60.8%; *p*-value: <0.01), cefepime (infants and young: 91.0%; preschool: 93.0%; school-aged: 92.3%; adolescents: 94.7%; *p*-value: 0.02), ceftaroline (infants and young: 85.9%; preschool: 88.5%; school-aged: 89.2%; adolescents: 92.0%; *p*-value: 0.01), and ceftriaxone (infants and young: 89.1%; preschool: 91.8%; school-aged: 90.7%; adolescents: 93.5%; *p*-value: <0.01), in which isolates derived from younger kids demonstrated lower susceptibility percentages. Most antimicrobial agents were found to have non-significant differences (*p*-value >0.05) in in vitro activity against UPEC across the different age groups.

There was a significantly greater number of UPEC recovered from female patients (*n* = 3011; 86.05%) than from male patients (*n* = 488; 13.95%). Gender data were not available for 12 isolates to include in the analysis. The data reveal gender-based variations in antibiotic susceptibility, with females generally showing higher susceptibility rates than males for most antibiotics: ciprofloxacin, levofloxacin, and trimethoprim–sulfamethoxazole exhibited a difference in their susceptibility rates of greater than 5% between female and male patients (*p*-value: <0.01). Other antibiotics with significant differences included aztreonam (F: 92.8% vs. M: 88.7%; *p*-value: <0.01), cefepime (F: 93.2% vs. M: 89.6%; *p*-value: <0.01), ceftaroline (F: 89.4% vs. M: 85.3%; *p*-value: 0.04), ceftazidime (F: 93.7% vs. M: 89.9%; *p*-value: <0.01), ceftriaxone (F: 91.8% vs. M: 87.5%; *p*-value: <0.01), piperacillin–tazobactam (F: 97.1% vs. M: 94.5%; *p*-value: <0.01), and tobramycin (F: 89.7% vs. M: 85.0%; *p*-value: <0.01) (Table 5).

Significant regional variations were observed in the susceptibility percentages for most antimicrobial agents; 12 antimicrobials (amoxicillin–clavulanic acid, ampicillin–sulbactam, cefepime, ceftaroline, ceftazidime, ceftriaxone, ciprofloxacin, doxycycline, levofloxacin, tetracycline, tobramycin, and trimethoprim–sulfamethoxazole) had a susceptibility difference greater than 10% between the nine U.S. census regions (Table 6).

The Middle Atlantic region (New Jersey, New York, and Pennsylvania) consistently presented with the lowest susceptibility rates compared to the other eight regions. The lowest susceptibility rates among the regions were observed for fifteen antibiotics (amikacin, aztreonam, cefepime, ceftaroline, ceftazidime, ceftolozane–tazobactam, ceftriaxone, ciprofloxacin, doxycycline, gentamicin, levofloxacin, minocycline, tetracycline, tobramycin, trimethoprim–sulfamethoxazole) from the Middle-Atlantic-region UPEC isolates.

Conversely, compared to the other census regions, isolates from the Mountain region (Arizona, Colorado, New Mexico, and Utah) were found to have the highest susceptibility rates for eight antibiotics (amoxicillin–clavulanic acid, ampicillin–sulbactam, ceftaroline, ceftriaxone, doxycycline, gentamicin, tetracycline, trimethoprim–sulfamethoxazole). The ceftazidime–avibactam, ceftolozane–tazobactam, colistin, imipenem, meropenem, and tigecycline susceptibility rates were the most regionally consistent (above 99%), with less than 1% difference in susceptibility percentages.

Over the 10-year study duration, a total of 752 (21.4%) and 339 (9.7%) UPEC isolates were identified as MDR and ESBL phenotypes, respectively. All the isolates were susceptible to imipenem and meropenem, except for one *E. coli* from the South Atlantic region (2019) that showed an imipenem MIC value of 2 mg/L (CLSI, intermediate). Carbapenemase genes were not detected in this isolate, although it carried *bla*_CMY-2_. Notably, three XDR-phenotype isolates were also identified, with one found in the East North Central region in 2018 and two in the Pacific region in 2017 and 2018.

**Figure 1:**
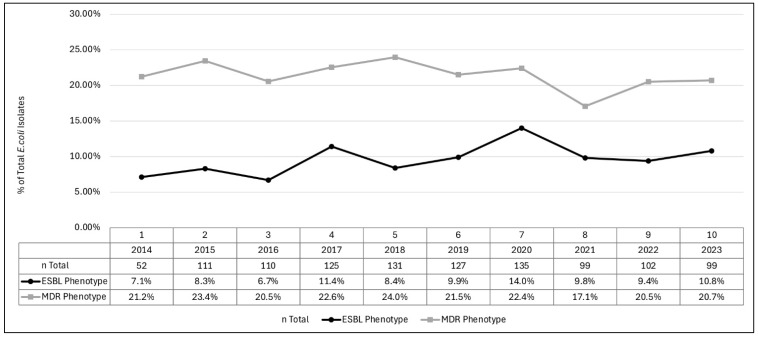
*E. coli* ESBL and MDR phenotype prevalence during 2014–2023 *. * ESBL: extended-spectrum beta-lactamase; MDR: multi-drug resistant (isolates non-susceptible to ≥1 agent in ≥3 antimicrobial categories).

**Figure 2:**
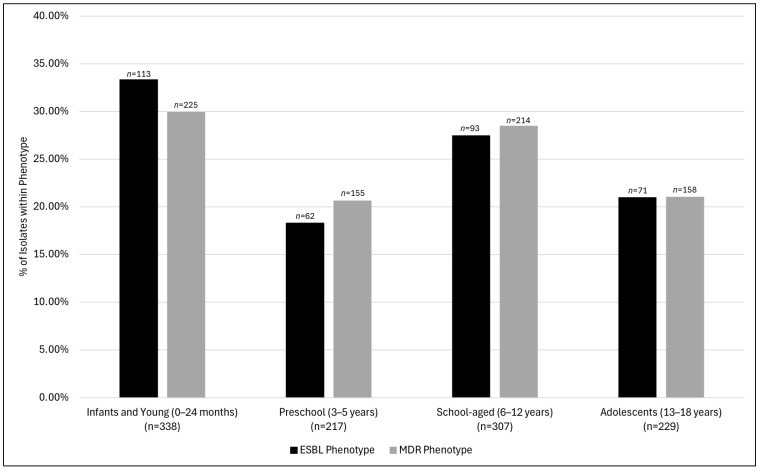
*E. coli* ESBL and MDR phenotypes by age group *. * ESBL: extended-spectrum beta-lactamase; MDR: multi-drug resistant (isolates non-susceptible to ≥1 agent in ≥3 antimicrobial categories).

**Figure 3:**
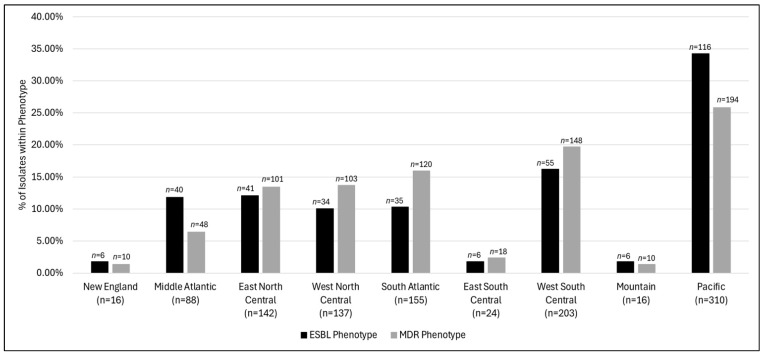
*E. coli* ESBL and MDR phenotypes by U.S. census division *. * ESBL: extended-spectrum beta-lactamase; MDR: multi-drug resistant (isolates non-susceptible to ≥1 agent in ≥3 antimicrobial categories).

A steady upward trend in ESBL prevalence in UPEC isolates was observed from 2014 (7.1%) to 2023 (10.8%) (*p*-value: 0.06). An increased prevalence was observed in 2020 (14.0%); however, the prevalence decreased to 9.8% and 9.4% during the years 2021 and 2022, respectively (Figure 1). The MDR prevalence remained relatively stable throughout the study duration from 2014 (21.2%) to 2023 (20.7%) (*p*-value: 0.56). Substantial variability was observed in the prevalence rates of the MDR as well as the ESBL phenotype among both age groups (MDR: *p*-value: 0.02 and ESBL: *p*-value: 0.06) and U.S. census regions groups (MDR and ESBL: *p*-value: <0.001) (Figure 2 and Figure 3). Similar trends were observed for the age category and geographical distribution for both MDR and ESBL phenotypes. The MDR and ESBL phenotypes were the most frequent among infants and young children (MDR: *n* = 225, 29.9%; ESBL: *n* = 113, 33.3%) followed by school-aged children (MDR: *n* = 214, 28.5%; ESBL: *n* = 93, 27.4%) (Figure 2). The frequency of the MDR (*n* = 194; 25.8%) and ESBL (*n* = 116; 34.2%) phenotypes in the Pacific region was much higher than that in the rest of the regions. The rates of the MDR and ESBL phenotypes in each of the remaining regions accounted for <20% of the respective resistant phenotype isolates. The MDR rates ranged from 6.4% in the Middle Atlantic (*n* = 48) to 19.7% in the West South Central (*n* = 148). The ESBL rates ranged from 10.0% in the West North Central region (*n* = 34) to 16.2% in the West South Central (*n* = 55). The rates of MDR and ESBL in the New England, East South Central, and Mountain regions each accounted for <3% of the respective resistant phenotype isolates.

## 4. Discussion

During the 10-year study duration from 2014 through to 2023, doxycycline (80.2% susceptible), tetracycline (76.2% susceptible), trimethoprim–sulfamethoxazole (69.7% susceptible), and ampicillin–sulbactam (55.7% susceptible) were found to be the least active agents against pediatric UPEC isolates. The current empiric treatment regimen for UTIs includes nitrofurantoin, first-generation cephalosporin (e.g., cephalexin), third-generation cephalosporin (e.g., ceftriaxone and ceftazidime), amoxicillin–clavulanate, and trimethoprim–sulfamethoxazole [4,6,7]. As per the Infectious Disease Society of America’s (IDSA’s) recommendation, any antimicrobial agent with antimicrobial resistance (AMR) >20% is not considered suitable to be used for empiric therapy [15]. Given these epidemiological findings, careful reevaluation should be considered against frequently used antibiotic agents like trimethoprim–sulfamethoxazole (69.7% susceptible) as a first-line empiric treatment option for UTIs in pediatric populations. A similar reduction in susceptibility rates and a recommendation against using these agents as first-line options was previously observed in pediatric uropathogen treatment in the United States [16,17,18]. It is important to note that the antimicrobial susceptibility trends in Europe vary widely across different regions. A study in Ireland recorded a similar lower susceptibility rate of <70% for trimethoprim–sulfamethoxazole [19], while a study from Spain found 22% of the pediatric UPEC isolates to be susceptible to trimethoprim–sulfamethoxazole [20]. Our study recorded significantly higher susceptibility rates among UPEC isolates for most antimicrobial agents than studies from Turkey and Taiwan [21,22]. In the Turkish study by Cag et al. (2021), the resistant rates for piperacillin–tazobactam (25.3% resistant), ceftazidime (37.1% resistant), ceftriaxone (36.7% resistant), cefepime (40.3% resistant), amikacin (12.1% resistant), ciprofloxacin (24.5% resistant), and amoxicillin–clavulanate (43.3%) were all higher than in our study (Table 2). Gentamicin (10.5% resistant) and trimethoprim–sulfamethoxazole (34% resistant) showed comparable resistance rates. Imipenem, meropenem, and nitrofurantoin had resistance rates <2%, similar to our findings [23]. The differences in the susceptibility rates may be due to geographical differences as well as the antibiotic usage practices in the patient population in different countries.

The susceptibility of UPEC isolates to most antimicrobial agents remained stable through the 10-year study duration. A single-center study from Ireland examined the six-year resistance trends for UPEC pediatric isolates and found a significant increase in susceptibility rates through the years for amoxicillin–clavulanic acid [19]. The opposite trend was observed in our data: 88.6% susceptible in 2014 to 83.4% susceptible in 2023 for amoxicillin–clavulanic acid. A retrospective cross-sectional study conducted at a hospital in Bahrain found that the percentage of *E. coli* resistance to ciprofloxacin increased over the study period from 9.52% in 2018 to 23.26% in 2020 [23]. We, however, observed an increased susceptibility trend for ciprofloxacin from 2014 (85.9% susceptible) to 2023 (90.4% susceptible). Several studies have been conducted in the adult population to understand the AMR trends among UPEC isolates [7,24,25,26]. There is little antibiotic susceptibility trend information among pediatric uropathogens [16]. Larger, surveillance-based pediatric AMR data among UPEC isolates may assist in optimizing empiric UTI treatment strategies in this patient population.

In our study, the UTI prevalence was higher in females. This aligns with several studies that have suggested that girls are more prone to urinary tract infections, likely due to their shorter urethras [1,2]. One study observed a higher number of urinary tract infections in male patients [13]. In our study, the isolates from male patients showed lower susceptibility to several antimicrobials. Ciprofloxacin, levofloxacin, and trimethoprim–sulfamethoxazole had over 5% lower susceptibility in males (*p*-value < 0.01). Other agents that demonstrated significant gender-based differences included aztreonam, cefepime, ceftaroline, ceftazidime, ceftriaxone, piperacillin–tazobactam, and tobramycin. This observation aligns with a case–control study that identified the male gender as an independent risk factor for ESBL-phenotype UPEC in children [27]. We also noted age-related differences, with isolates from younger children showing lower susceptibility to several antimicrobials including ampicillin–sulbactam (*p*-value: <0.01), cefepime (*p*-value: 0.02), ceftaroline (*p*-value: 0.01), and ceftriaxone (*p*-value: <0.01). Similar observations have been made regarding differences in the patient age and gender among pediatric uropathogens in the United States and in Taiwan [16,22].

The susceptibility patterns differed markedly across the country, with lower susceptibility rates generally observed in the Middle Atlantic region for fifteen antimicrobials (amikacin, aztreonam, cefepime, ceftaroline, ceftazidime, ceftolozane–tazobactam, ceftriaxone, ciprofloxacin, doxycycline, gentamicin, levofloxacin, minocycline, tetracycline, tobramycin, and trimethoprim–sulfamethoxazole), while higher susceptibility rates were generally observed in the Mountain region for eight antimicrobials (amoxicillin–clavulanic acid, ampicillin–sulbactam, ceftaroline, ceftriaxone, doxycycline, gentamicin, tetracycline, and trimethoprim–sulfamethoxazole). The resistant phenotypes (ESBL and MDR) also varied by region, with the highest prevalence seen in the Pacific region. Regional variation, mostly in the adult population, has been well documented in the literature [7,25,28,29]. The risk factors contributing to this disparity may include regional differences in patient demographics, differences in antibiotic prescribing patterns, climate, international travel, and local public health initiatives. The antimicrobial usage data by geographical region among both humans and agricultural sectors in the United States are not well documented; therefore, direct correlation is not possible [29]. However, studies have suggested that areas with higher rates of antibiotic prescriptions tend to have higher levels of resistance [29,30,31]. The regional heterogenicity of the AMR phenotype distribution has also been attributed to the heterogeneous distribution of distinct resistance clones with shared genetic resistance elements, e.g., plasmids and integrons. [29]. A previous study performing the epidemiological analysis of UPEC isolates suggested that *E. coli* clonal type A (CGA) was found to be associated with trimethoprim–sulfamethoxazole resistance; this strain was found to be more prevalent specifically in Los Angeles, CA, than in other U.S. centers including other Pacific-region centers [32]. A surveillance study on gestational UPEC isolates derived from pregnant women with pyelonephritis observed an association of ampicillin resistance with isolates having a *dra* gene cluster, which encodes for colonization and invasive capacity [33]. The UPEC ST131 clone is the most dominant globally disseminated UPEC clone; a recent study found that the *dr* adhesion genes were frequently found among the ST131 clones [34]. The prevalence of these genes was found to be the highest among Clade A (36.5%) followed by Clade C (28.4%) and B (19.2%); within Clade C the prevalence of *dr* adhesion genes was significantly higher (*p* < 0.0001) among the subclade C2, which has an MDR phenotype [34]. These findings highlight the importance of tailoring antibiotic stewardship programs to address the specific needs and national/regional surveillance studies to understand resistance patterns/clonal spread in each region, which could ultimately improve the effectiveness of UTI treatments and curb the spread of resistant *E. coli* strains.

The ESBL rates in *E. coli* isolates have significantly increased over the years for both pediatric and adult populations [27,35,36]. Our study also shows a steady increase in ESBL prevalence in UPEC over the study period (7.1% in 2014 to 10.8% in 2023). A decline in ESBL prevalence following the onset of the COVID-19 pandemic was observed (14.0% in 2020 as compared to 9.8% and 9.4% during the years 2021 and 2022, respectively). A similar observation was made in other countries [37]. The declines in ESBL prevalence suggest that pandemic-related factors such as changes in antibiotic use and enhanced infection control measures may have influenced resistance patterns. Risk factor analysis for UPEC UTI in children found male gender, history of urology care, and previous antibiotic exposure as risk factors for ESBL-UPEC UTI in children [27]. Another study covering all age groups (0–65 years) documented an increased prevalence of ESBL-UPEC over more than a 6-year period and evaluated the corresponding risk factors for both community- and healthcare-associated bacteriuria [38]. An average increase of 0.9% and 2.3% per year was observed for community- and healthcare-onset ESBL-UPEC episodes, respectively. Male gender and race were associated with community-onset, and only male gender was associated with healthcare-onset ESBL UPEC [38].

By leveraging the robust dataset from the SENTRY program, this study aimed to provide a detailed overview of the susceptibility patterns of UPEC from pediatric patients against commonly used antibiotics. The extended study period (2014–2023) with the substantial overall sample size (*n* = 3511) offers a robust temporal analysis. Additionally, the inclusion of multiple demographic factors provides a comprehensive comparison of antibiotic susceptibility patterns for a range of antibiotics. The study limitations include the retrospective nature of this study. There were missing data for amoxicillin–clavulanic acid (years: 2015 and 2016), ceftaroline (years: 2021–2023), and nitrofurantoin (years: 2014–2016) since susceptibility testing was not performed on those agents for those years as depicted in Table 3. The varying sample size each year and by region (lower sample size for year 2014 and New England region) may also influence the comparative susceptibility results. There could be variations in data reporting across different regions and hospitals although all the medical centers were required to send consecutive isolates. Additionally, the lack of clinical data (e.g., differentiation between uUTI and cUTI, isolates, sample-collection setting: inpatient vs. outpatient units, and prior antimicrobial therapies) introduces the possibility of selection bias, which cannot be entirely ruled out. Since cultures are not routinely performed, the patient population in this study may be skewed toward individuals with difficult-to-treat, recurrent, and/or complicated UTIs. These cases are more likely to undergo follow-up culture and/or susceptibility testing. As a result, the isolates in this study may over-represent non-susceptible or resistant UTI strains to some degree.

Overall, these data highlight the importance of continuous national and regional studies to understand antimicrobial susceptibility patterns in *E. coli* pediatric UTI isolates. Lower observed susceptibility rates (<80%) for certain antibiotics may warrant revised recommendations regarding agents that are currently used as first-line empiric treatment options. Ongoing monitoring and adaptive empiric treatment strategies are essential to address evolving resistance patterns.

## Figures and Tables

**Table 1:** Patient demographics by U.S. census region ^†^.

Demographics		U.S. Census Region									Total *n* (%)
		New England (*n* = 68)	Middle Atlantic (*n* = 135)	East North Central (*n* = 573)	West North Central (*n* = 527)	South Atlantic (*n* = 517)	East South Central (*n* = 89)	West South Central (*n* = 704)	Mountain (*n* = 105)	Pacific (*n*= 793)	
Sex *	Male (*n* = 488)	4	21	64	57	91	8	98	9	136	488 (14.0)
	Female (*n* = 3011)	64	114	506	470	419	81	605	96	656	3011 (86.1)
Age	Infants and young (0–24 months) (*n* = 970)	7	40	137	136	140	27	224	16	243	970 (27.6)
	Preschool (3–5 years) (*n* = 703)	18	23	103	112	91	12	148	15	181	703 (20.0)
	School-aged (6–12 years) (*n* = 955)	18	32	172	149	130	19	185	24	226	955 (27.2)
	Adolescents (13–18 years) (*n* = 883)	25	40	161	130	156	31	147	50	143	883 (25.2)

* Gender data were not available for 12 isolates. ^†^ Geographic regions were based on the U.S. census divisions described by the Centers for Disease Control and Prevention: https://www.cdc.gov/nchs/hus/sources-definitions/geographic-region.htm (accessed on 1 September 2024).

**Table 2:** Overall antimicrobial activity of common agents tested against uropathogenic *E. coli* recovered from the pediatric population in the U.S. during 2014–2023.

Antimicrobial	Susceptibility Breakpoint ^a^	% Susceptible	MIC_50_ (µg/mL)	MIC_90_ (µg/mL)
Amikacin	≤4	93.7	2	4
Amoxicillin–clavulanic acid	≤8/4	83.2	4	16
Ampicillin–sulbactam	≤8/4	55.7	8	>32
Aztreonam	≤4	92.2	≤0.12	0.5
Cefepime	≤2	92.7	0.06	≤0.5
Ceftaroline	≤0.5	88.9	0.06	1
Ceftazidime	≤4	93.2	0.12	1
Ceftazidime–avibactam	≤8/4	100	0.06	0.12
Ceftolozane–tazobactam	≤2/4	99.6	0.12	0.25
Ceftriaxone	≤1	91.2	≤0.06	0.25
Ciprofloxacin	≤0.25	85.4	≤0.03	>4
Colistin ^a^	≤2	99.8	0.25	0.25
Doxycycline	≤4	80.2	1	>8
Gentamicin	≤2	89.3	≤1	8
Imipenem	≤1	99.9	≤0.12	≤0.12
Levofloxacin	≤0.5	87.0	0.03	>4
Meropenem	≤1	100	≤0.05	0.03
Minocycline	≤4	91.1	0.5	4
Nitrofurantoin	≤32	98.1	16	32
Piperacillin–tazobactam	≤8/4	96.7	2	4
Tetracycline	≤4	76.2	1	>16
Tigecycline ^a^	≤2	99.9	0.12	0.25
Tobramycin	≤2	89.1	1	4
Trimethoprim–sulfamethoxazole	≤2/38	69.7	≤0.5	>8

^a^ Susceptible breakpoint as published by the CLSI M100 (2024) guideline, except for colistin and tigecycline, for which the EUCAST (2024) and FDA (2024) susceptible criteria were applied, respectively.

**Table 3:** Yearly activity of common agents tested against uropathogenic *E. coli* recovered from the pediatric population in the U.S. during 2014–2023.

Antimicrobial	Percent Susceptibilities Through the Years										*p*-Value ^a^
	2014 (*n* = 184)	2015 (*n* = 350)	2016 (*n* = 404)	2017 (*n* = 368)	2018 (*n* = 405)	2019 (*n* = 405)	2020 (*n* = 371)	2021 (*n* = 369)	2022 (*n* = 341)	2023 (*n* = 314)	
Amikacin	98.4	96.0	93.8	91.3	90.6	92.3	90.6	94.0	97.4	96.5	<0.01
Amoxicillin–clavulanic acid	88.6	-	-	79.4	82.2	80.7	84.0	86.2	82.3	83.4	0.21
Ampicillin–sulbactam	59.6	55.4	55.7	53.5	52.8	56.0	52.3	58.3	58.9	56.7	0.59
Aztreonam	92.9	94.0	94.6	91.0	93.6	92.3	87.9	91.6	92.4	91.7	0.07
Cefepime	95.1	94.9	95.0	91.8	93.3	92.3	87.9	91.9	92.7	93.0	0.02
Ceftaroline	91.8	90.9	91.1	86.4	88.6	86.7	87.9	-	-	-	0.17
Ceftazidime	94.0	94.3	94.6	91.8	94.3	93.3	89.2	93.2	93.8	93.3	0.21
Ceftazidime–avibactam	100	100	100	100	100	100	100	100	100	100	-
Ceftolozane–tazobactam	100	98.9	99.3	99.5	100	100	100	100	99.7	99.0	0.06
Ceftriaxone	92.9	92.3	93.6	90.2	92.8	90.6	87.1	91.1	90.6	91.1	0.13
Ciprofloxacin	85.9	84.6	87.4	84.8	84.9	84.7	81.7	85.1	87.5	90.4	0.26
Colistin	100	100	100	100	99.5	99.8	100	99.7	99.1	99.7	0.26
Doxycycline	81.5	79.4	79.7	77.3	75.3	77.0	80.2	84.0	83.9	85.6	0.13
Gentamicin	88.0	89.4	91.1	88.3	89.6	91.3	88.1	91.6	88.3	85.6	0.26
Imipenem	100	100	100	100	100	99.8	100	100	100	100	1.00
Levofloxacin	88.0	86.9	88.1	87.5	87.9	86.1	83.8	87.5	85.9	88.9	0.76
Meropenem	100	100	100	100	100	100	100	100	100	100	-
Minocycline	92.4	92.9	88.9	89.6	88.1	89.7	88.9	94.0	93.2	94.3	0.09
Nitrofurantoin	-	-	-	97.6	98.3	97.1	97.3	99.3	98.4	98.7	0.49
Piperacillin–tazobactam	97.3	97.1	95.0	95.9	97.8	94.6	97.0	98.4	97.1	97.8	0.07
Tetracycline	79.9	72.3	77.2	73.9	72.2	74.5	76.8	80.0	77.6	79.5	0.33
Tigecycline	100	100	100	100	100	99.5	100	100	100	100	0.21
Tobramycin	88.6	88.9	91.6	88.0	89.4	90.6	87.9	90.5	88.0	86.0	0.43
Trimethoprim–sulfamethoxazole	69.9	68.9	68.3	67.7	65.7	69.4	71.4	73.4	72.1	70.9	0.48

“-” Susceptibility data not available. ^a^ Significant difference was considered when *p*-value < 0.05.

**Table 4:** Activity of common agents tested against uropathogenic *E. coli* recovered from the pediatric population in the U.S. during 2014–2023 by age group.

Antimicrobial	Infants and Young (*n* = 970)	Preschool (*n* = 703)	School-Aged (*n* = 955)	Adolescents (*n* = 883)	*p*-Value ^a^
Amikacin	92.3	93.9	94.8	94.1	0.15
Amoxicillin–clavulanic acid	81.9	83.5	84.1	83.4	0.79
Ampicillin–sulbactam	52.2	53.6	56.0	60.8	<0.01
Aztreonam	91.1	92.5	91.6	93.8	0.16
Cefepime	91.0	93.0	92.3	94.7	0.02
Ceftaroline	85.9	88.5	89.2	92.0	0.01
Ceftazidime	91.8	93.5	92.9	94.8	0.07
Ceftazidime–avibactam	100	100	100	100	-
Ceftolozane–tazobactam	99.4	99.4	99.8	99.9	0.28
Ceftriaxone	89.1	91.8	90.7	93.5	0.01
Ciprofloxacin	85.9	86.7	84.2	85.1	0.56
Colistin	99.8	99.9	100	99.4	0.07
Doxycycline	79.6	80.7	78.8	82.1	0.50
Gentamicin	89.1	88.1	89.1	90.8	0.33
Imipenem	100	99.9	100	100	0.20
Levofloxacin	87.2	88.3	85.4	87.4	0.35
Meropenem	100	100	100	100	-
Minocycline	90.1	92.0	90.2	92.4	0.34
Nitrofurantoin	98.8	98.3	98.1	97.3	0.44
Piperacillin–tazobactam	96.1	96.0	96.9	97.9	0.10
Tetracycline	75.4	74.8	75.1	79.2	0.23
Tigecycline	99.9	99.9	100	100	0.57
Tobramycin	88.0	87.8	89.3	90.9	0.13
Trimethoprim–sulfamethoxazole	68.3	65.5	68.9	75.4	<0.01

^a^ Significant difference was considered when *p*-value < 0.05.

**Table 5:** Activity of common agents tested against uropathogenic *E. coli* recovered from the pediatric population in the U.S. during 2014–2023 by patient gender.

Antimicrobial	Female (*n* = 3011)	Male (*n* = 488)	*p*-Value ^a^
Amikacin	94.0	91.8	0.07
Amoxicillin–clavulanic acid	83.6	81.0	0.28
Ampicillin–sulbactam	56.2	52.5	0.13
Aztreonam	92.8	88.7	<0.01
Cefepime	93.2	89.6	0.01
Ceftaroline	89.4	85.3	0.04
Ceftazidime	93.7	89.9	<0.01
Ceftazidime–avibactam	100	100	-
Ceftolozane–tazobactam	99.7	99.3	0.24
Ceftriaxone	91.8	87.5	<0.01
Ciprofloxacin	86.2	79.9	<0.01
Colistin	99.8	99.6	0.31
Doxycycline	80.6	77.9	0.25
Gentamicin	89.6	87.7	0.21
Imipenem	99.9	100	1.00
Levofloxacin	87.8	81.8	<0.01
Meropenem	100	100	-
Minocycline	90.7	93.2	0.13
Nitrofurantoin	98.3	96.7	0.09
Piperacillin–tazobactam	97.1	94.5	0.01
Tetracycline	76.5	74.6	0.46
Tigecycline	99.9	100	1.00
Tobramycin	89.7	85.0	<0.01
Trimethoprim–sulfamethoxazole	70.9	62.4	<0.01

^a^ Significant difference was considered when *p*-value < 0.05.

**Table 6:** Activity of common agents tested against uropathogenic *E. coli* recovered from the pediatric population in the U.S. during 2014–2023 by U.S. census division.

Antimicrobial	New England (*n* = 68)	Middle Atlantic (*n* = 135)	East North Central (*n* = 573)	West North Central (*n* = 527)	South Atlantic (*n* = 517)	East South Central (*n* = 89)	West South Central (*n* = 704)	Mountain (*n* = 105)	Pacific (*n* = 793)	*p*-Value ^a^
Amikacin	95.6	92.6	94.8	93.2	94.2	93.3	94.0	94.3	92.8	0.91
Amoxicillin–clavulanic acid	83.7	82.5	85.6	83.1	78.6	83.1	84.1	90.9	84.5	0.37
Ampicillin–sulbactam	70.6	56.3	58.1	57.3	51.7	56.2	51.8	77.1	54.5	<0.01
Aztreonam	91.2	75.6	95.3	95.1	94.6	93.3	93.9	95.2	87.4	<0.01
Cefepime	92.6	75.6	95.6	94.9	95.9	93.3	94.1	95.2	88.1	<0.01
Ceftaroline	87.5	64.3	90.6	91.4	90.8	87.1	91.9	92.8	86.9	<0.01
Ceftazidime	92.6	79.3	95.3	95.8	95.7	95.5	95.3	95.2	88.1	<0.01
Ceftazidime–avibactam	100	100	100	100	100	100	100	100	100	-
Ceftolozane–tazobactam	100	99.1	99.2	99.8	99.6	100	99.8	100	99.6	0.69
Ceftriaxone	91.2	70.4	94.2	94.3	94.2	93.3	93.2	95.2	85.9	<0.01
Ciprofloxacin	93.8	68.2	87.4	87.7	86.4	89.9	85.9	90.7	82.0	<0.01
Colistin	100	100	99.7	99.2	99.6	100	100	100	100	0.15
Doxycycline	80.0	68.6	82.5	82.7	77.9	82.1	81.2	89.4	78.6	0.06
Gentamicin	92.6	85.2	89.4	89.7	90.3	87.6	89.0	93.3	88.6	0.65
Imipenem	100	100	100	100	99.81	100	100	100	100	0.27
Levofloxacin	95.6	69.6	89.4	89.4	87.6	89.9	86.5	93.3	84.9	<0.01
Meropenem	100	100	100	100	100	100	100	100	100	-
Minocycline	94.0	86.0	92.9	92.5	90.3	94.6	91.4	89.4	89.1	0.31
Nitrofurantoin	97.6	98.1	98.4	99.2	95.9	100	98.1	100	98.5	0.17
Piperacillin–tazobactam	98.5	96.3	96.7	96.8	96.1	100	96.9	98.1	96.3	0.73
Tetracycline	78.0	61.6	77.8	80.0	73.5	78.6	77.2	89.4	73.7	0.01
Tigecycline	100	100	100	100	100	100	99.9	100	99.9	1.00
Tobramycin	92.6	80.7	90.6	88.9	91.5	88.8	88.8	92.4	87.4	0.03
Trimethoprim–sulfamethoxazole	80.9	60.7	75.0	73.4	67.7	69.7	63.3	81.9	69.2	<0.01

^a^ Significant difference was considered when *p*-value <0.05.

## Data Availability

The datasets analyzed can be readily accessed. The web links to the SENTRY Antimicrobial Surveillance Program and the publicly available database are https://www.jmilabs.com/sentry-surveillance-program/ and https://sentry-mvp.jmilabs.com/ (accessed on 1 September 2024), respectively. Further inquiries can be directed to the corresponding author.
